# Do Specific Craniomaxillofacial Features Correlate with Psychological Distress in Adult Pretreatment Orthodontic Patients? A Cephalometric Study

**DOI:** 10.1155/2022/9694413

**Published:** 2022-05-05

**Authors:** Chu-Qiao Xiao, Yi-Dan Wan, Zhe-Bin Yan, Ya-Qi Li, Pei-Di Fan, Qiao-Yu Cheng, Xin Xiong

**Affiliations:** ^1^National Clinical Research Center for Oral Diseases, State Key Laboratory of Oral Diseases, Department of Orthodontics, West China Hospital of Stomatology, Sichuan University, Chengdu, Sichuan, China; ^2^Department of Nursing, West China Hospital of Stomatology, Sichuan University, Chengdu, Sichuan, China

## Abstract

**Purpose:**

To explore the relationship between craniomaxillofacial features and psychological distress among adult pretreatment orthodontic patients.

**Methods:**

A group of 190 patients (95 males and 95 females) was included. Questionnaires including the Kessler psychological distress scale (K10) were sent to patients, and cephalograms were collected. Patients were divided into two groups according to K10 score: psychological distress group (score ≥ 20) and no psychological distress group (score < 20). Nineteen hard tissue and thirteen soft tissue parameters were traced on cephalograms to characterize the craniomaxillofacial features.

**Results:**

There was no significant difference in gender or age distribution between the two groups. Male patients with psychological distress showed statistically significantly larger anterior facial height (AFH) (126.62 mm vs. 120.97 mm), upper lip length (25.11 mm vs. 23.26 mm), and smaller overbite (1.21 mm vs. 2.75 mm) than patients without psychological distress. Male patients with hyperdivergent pattern and open bite were more likely to have psychological distress. None of the parameters showed statistical differences across groups in females. Frankfort-mandibular plane angle (*r* = 0.235), Bjork's sum (*r* = 0.311), AFH (*r* = 0.322), overbite (*r* = −0.238), AFH/posterior facial height (*r* = 0.251), and upper anterior facial height (UAFH)/lower anterior facial height (LAFH) (*r* = −0.230) were correlated with K10 score in males. After adjusting gender and age, the AFH (*B* = 0.147) and UAFH/LAFH (*B* = −14.923) were significantly related with the K10 score.

**Conclusion:**

Psychological distress was mainly correlated with hyperdivergent pattern, open bite, and larger lower anterior facial height proportion in pretreatment orthodontic patients. Orthodontists should be aware of the possible underlying psychological distress in patients with specific craniomaxillofacial features. Clinical assessment of psychological distress may need to take into account gender differences in patients.

## 1. Introduction

Orthodontic patients with craniomaxillofacial deformities or unattractive dentition are more likely to experience psychological distress due to decreased self-esteem and confidence [1, 2]. Around 44% to 63% of orthodontic patients declare that their facial appearance problems adversely affect their personal or social life [3]. While experiencing a reduction of facial self-perception, patients generally develop higher levels of anxiety, depression, and social isolation [4, 5]. It is of great significance for orthodontists to take these psychological problems into account because patients with high levels of psychological distress might tend to overrate the pain they have experienced, exhibit more negative emotions, be averse to follow-up treatment, and be dissatisfied with the treatment effects [1, 6, 7]. Therefore, evaluation of patients' psychological status can enable orthodontists to identify potential problems and avoid medical disputes at an early stage [8].

Some studies have suggested that the psychological problems of orthodontic patients may be associated with their craniomaxillofacial features. Due to the influences of self-awareness, family, and society, patients with craniofacial deformities may suffer reduced quality of life and more negative emotions such as anxiety and depression [9, 10]. However, a study by Kovalenko et al. found that only serious facial deformities were associated with mental instability, introversion, and unsociability [11]. Some researchers have even suggested that patients who are dissatisfied with their facial appearance have no special psychological problems [12]. The relationship between craniomaxillofacial morphology and patients' psychological status is still unclear. Moreover, hard tissue and soft tissue structures might affect craniomaxillofacial features and facial aesthetics jointly [13], and improving facial aesthetics is one of the main purposes of patients pursuing orthodontic treatment [14]. Therefore, it would be more appropriate to evaluate craniomaxillofacial features by considering hard tissue and soft tissue structures together.

In this study, the psychological distress status of adult pretreatment orthodontic patients was assessed by the Kessler psychological distress scale (K10), and both hard tissue and soft tissue structures were evaluated on lateral cephalograms. The null hypothesis was that there would be no specific craniomaxillofacial features correlate with psychological distress in adult pretreatment orthodontic patients.

## 2. Materials and Methods

### 2.1. Subjects

The study consecutively distributed questionnaires to adult pretreatment orthodontic patients from the Department of Orthodontics, West China Hospital of Stomatology, Sichuan University, from September to October 2021. The inclusion criteria were (i) able to comprehend and fill out all questions in the questionnaire; (ii) having clear lateral cephalogram data from the first visit; and (iii) age over 18 years. The exclusion criteria were (i) having a previous history of orthodontic or orthognathic treatment; (ii) having a diagnosis of systemic, metabolic, neurological, or immune disorder or disease; (iii) having a history of head, neck, or oral trauma, orofacial or plastic surgery, and other diseases which may cause craniomaxillofacial deformity; and (iv) questionnaire not completed or obviously filled in randomly.

### 2.2. Questionnaire

**Figure 1:**
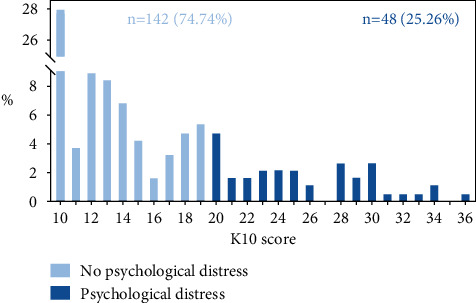
Distribution of K10 scores in overall study sample (*n* = 190).

The questionnaire included demographic information and a Chinese version of the K10. The K10 is a 10-item scale to measure the frequency of negative psychological symptoms such as nervousness, hopelessness, and worthlessness experienced in the past four weeks and consists of two parts: anxiety symptoms (4 items) and depression symptoms (6 items) [15]. Responses to each item were recorded on a 5-point Likert scale ranging from 1 (none of the time) to 5 (all of the time) and were summed to produce a total score ranging from 10 to 50. Patients were divided into two groups according to the K10 score: psychological distress group (score ≥ 20) and no-distress group (score < 20) (Figure 1) [16, 17]. In addition, the K10 score can also be considered as a continuous variable, with a higher score indicating greater symptoms of psychological distress [17].

### 2.3. Ethical Statement

The protocol of this cross-sectional study was approved by West China Hospital of Stomatology of Sichuan University Ethics Committee (Approval no. WCHSIRB-D-2021-430) and conducted in accordance with the Declaration of Helsinki. All participants were informed about the purpose of this study. Written and signed consent was obtained from every study participant.

### 2.4. Cephalometric Analysis

All cephalograms were performed at the Department of Medical Imaging, West China Hospital of Stomatology, Sichuan University, by the same trained professional. Every subject was instructed to adopt a natural head position with teeth in centric occlusion during radiography.

**Figure 2:**
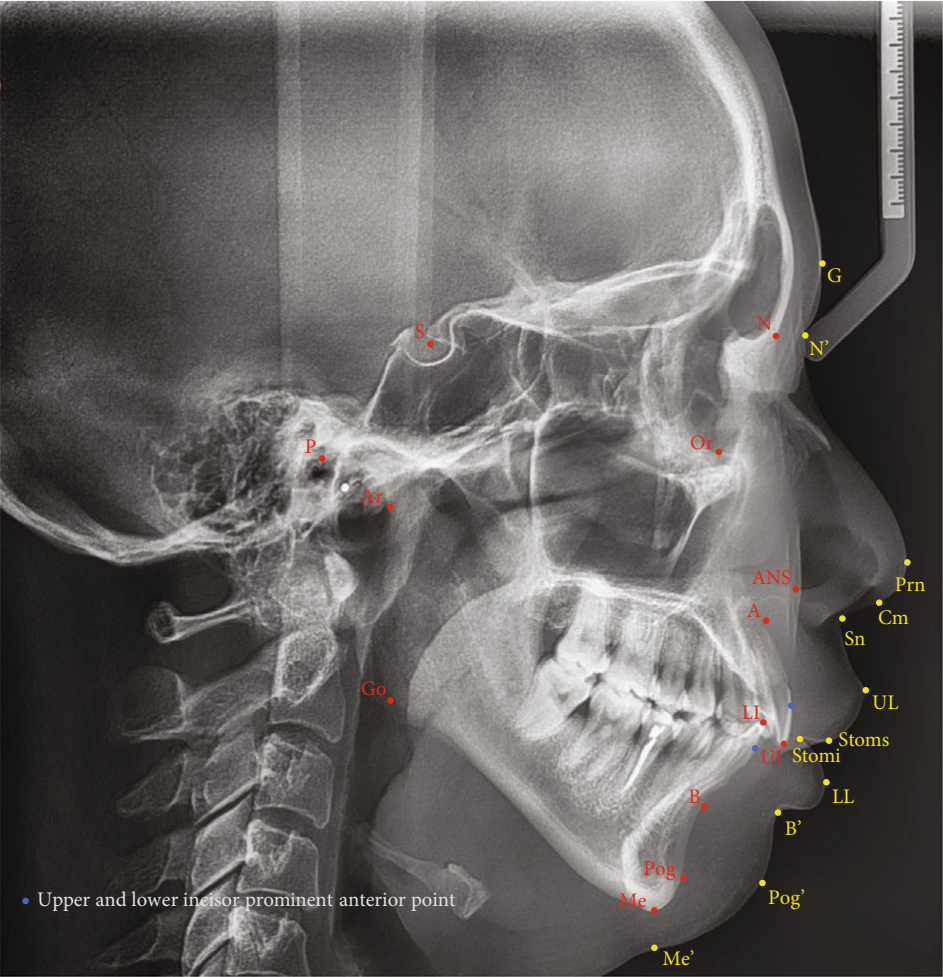
Hard tissue and soft tissue landmarks on lateral cephalogram. Red and blue points show hard tissue landmarks on lateral cephalogram; yellow points show soft tissue landmarks. N: nasion; S: sella; P: porion; Or: orbitale; Ar: articulare; ANS: anterior nasal spine; A: subspinale; UI: upper incisor; LI: lower incisor; B: supramental; Pog: pogonion; Me: menton; Go: gonion; G: glabella; N': nasion of soft tissue; Prn: pronasale; Cm: columella; Sn: subnasale; UL: upper lip; Stoms: stomion superius; Stomi: stomion inferius; LL: lower lip; B': soft tissue B point; Pog': pogonion of soft tissue; Me': menton of soft tissue.

In this study, 19 hard tissue parameters (6 angles, 11 linear distances, and 2 proportions) and 13 soft tissue parameters (3 angles, 9 linear distances, and 1 proportion) were measured and analyzed by the same investigator using Uceph software (Chengdu Yaxun, Chengdu, China), including 13 hard tissue and 14 soft tissue landmarks (Figure 2). Hard tissue parameters included the following: (i) angular measurements: ANB (A-N-B), Frankfort-mandibular plane angle (FMA, FH-OP), saddle angle (N-S-Ar), articular angle (S-Ar-Go), gonial angle (Ar-Go-Me), and Bjork's sum (sum of saddle, articular, and gonial angle); (ii) linear measurements: ramus height (Ar-Go), mandibular body length (Go-Me), anterior facial height (AFH, N-Me), posterior facial height (PFH, S-Go), anterior cranial base length (S-N), posterior cranial base length (S-Ar), wits (connect the cusp tips of first molar and second premolar as functional occlusal plane (FOP), draw a perpendicular from point A and point B to the FOP; the distance between the two foot points is wits), overjet (horizontal overlap of UI and LI), overbite (vertical overlap of UI and LI), lower anterior facial height (LAFH, ANS-Me), and upper anterior facial height (UAFH, ANS-N); and (iii) proportion: AFH/PFH (N-Me/S-Go), UAFH/LAFH (ANS-N/ANS-Me). Soft tissue parameters included the following: (i) angular measurements: facial convexity (G-Sn-Pog'), nasolabial angle (Cm-Sn-UL'), and nasal prominence (Prn-N'-Sn); (ii) linear measurements: upper lip length (stoms-Sn), lower lip length (stomi-B'), lower lip to E plane (horizontal distance between LL and esthetic plane), upper lip to E plane (horizontal distance between UL and esthetic plane), lower lip thickness (LL to lower incisor prominent anterior point), upper lip thickness (UL to upper incisor prominent anterior point), soft tissue chin thickness (Pog-Pog'), N'-Sn, and Sn-Me'; and (iii) proportion: N'-Sn/Sn-Me' [18, 19].

**Figure 3:**
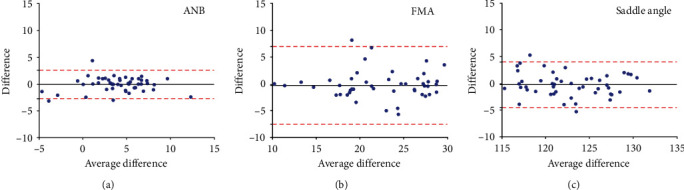
Bland-Altman plots demonstrating the bias for cephalometric variables: (a) ANB, (b) FMA, and (c) saddle angle. Only three parameters were shown in this figure. The two dashed red lines are the 95% limits of test-retest agreement. The black lines show the mean of the differences, which are close to 0, indicating low test-retest bias. FMA: Frankfort-mandibular plane angle.

To test intrarater reliability, after completing all the cephalometric measurements, 50 cephalograms were randomly selected and remeasured two weeks later by the same investigator. Then, the Bland-Altman analysis was performed for all cephalometric parameters. For each cephalometric parameter, the test-retest difference in cephalometric measurement was plotted against the average difference. Of the thirty two parameters, the first three Bland-Altman plots were demonstrated (Figure 3). Test-retest bias ranged from 0.06 to 0.55. All *P* values were greater than 0.05, indicating none of the biases calculated was significant.

### 2.5. Cephalometric Parameter Stratification

Stratification analysis was conducted on cephalometric parameters in accordance with previous studies [20–22]. Patients were divided into skeletal class I (0° ≤ ANB ≤ 4°), class II (ANB > 4°), and class III (ANB < 0°) according to ANB. Patients were categorized as hypodivergent (FMA < 22 , normodivergent (22 ≤ FMA  ≤ 28 , and hyperdivergent (FMA > 28°) according to FMA. Additionally, patients were divided into crossbite (overjet < 1 mm), normal (1 mm ≤ overjet ≤ 3 mm), and deep overjet (overjet > 3 mm) groups and into open bite (overbite < 1 mm), normal (1 mm ≤ overbite ≤ 3 mm), and deep overbite (overbite > 3 mm) groups.

### 2.6. Statistical Analysis

The sample size was computed by using G∗power (version 3.1.9, Germany) considering *α* = 0.05 and power = 0.80. Based on our previous cross-sectional studies [23], the mean value for anterior facial height in pretreatment orthodontic patients was 116.07 ± 7.84 mm compared with 120.10 ± 8.92 mm in the control group. We assumed that the sampling ratio (patients with psychological distress/patients without psychological distress) was 0.25 and standard deviation within each group = 7 mm. The analysis revealed that effect size of *d* was 0.57, and 176 subjects were necessary to perform the study. A total of 190 subjects were included in this study.

Data analyses were carried out using SPSS 21.0 software (IBM, New York, USA). Quantitative data are expressed as mean ± standard deviation, and qualitative data are expressed as quantity and frequency. The Shapiro–Wilk test was used to analyze the normality of age, K10 score, and cephalometric parameters. Nonnormally distributed data (K10 score, age, ANB, wits appraisal, overjet, and facial convexity parameters) between the no-distress and psychological distress groups were analyzed by the Mann-Whitney *U* test. Normally distributed cephalometric parameters data were analyzed by independent samples *t*-test. The chi-square test or Fisher test was used to examine the differences in categorical variables (gender and cephalometric parameter stratification) between the two groups. The Spearman correlation analysis was used to correlate K10 score and cephalometric parameters. Correlations were interpreted as follows: weak correlation, *r* < 0.30; moderate correlation, 0.30 < *r* < 0.50; and strong correlation, *r* > 0.50 [24]. And the results of correlation analysis were visualized by scatter plots. The relationship between the K10 score and the cephalometric parameters was determined using multivariate linear regression test, adjusted for gender and age. The dependent variables were K10 score. The *P* values of independent *t*-test and correlation analysis were adjusted with the Benjamini and Hochberg method. The test level was *α* = 0.05, and adjusted *P* values < 0.05 indicated statistically significant differences.

## 3. Results

One hundred and ninety patients were included in this study. There were 46 patients in the psychological distress group, including 22 males (11.58%) and 26 females (13.68%), and 142 patients in the no-distress group, including 73 males (38.42%) and 69 females (36.32%). There was no significant difference between the groups in terms of gender or age distribution (Table 1).

**Figure 4:**
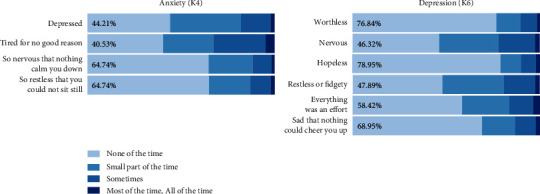
Frequencies of K10 responses in overall patient sample. Percentage indicates the frequency of “none of the time” responses to the indicated item.

In the four items in the K10 indicating anxiety, the most common symptoms (i.e., responses other than “none of the time”) were *feel tired for no good reason* (59.47%) and *feel depressed* (55.79%); in the six items indicating depression, the most common symptoms were *feel nervous* (53.68%) and *feel restless or fidgety* (52.11%) (Figure 4).

Overall, no significant differences were observed after adjusting *P* values for multiple testing (Supplemental Table 1). The subgroup analysis showed that there were significant differences across groups in hard and soft tissue cephalometric parameters in males. Compared to nondistressed participants, among the hard tissue measurements, male patients in the psychological distress group had larger AFH (*t* = −4.621, adjusted *P* = 0.001) as well as a smaller overbite (*t* = 3.332, adjusted *P* = 0.016). Soft tissue measurements showed longer upper lip length (*t* = −3.052, adjusted *P* = 0.032) in the male psychological distress group (Table 2). Among female patients, no statistically significant difference was found between the two groups in any parameter (Supplemental Table 2).

Further, we found that male patients with a hyperdivergent pattern (*P* = 0.029) and open bite (*P* = 0.010) were more likely to have psychological distress. Cephalometric parameter stratification analysis did not show any statistical difference in females (Table 3).

**Figure 5:**
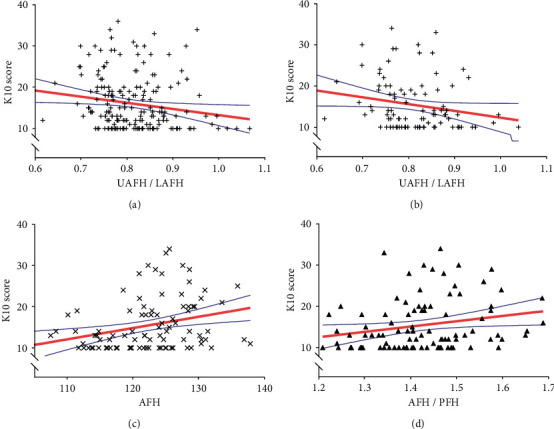
Scatterplot for the correlations between K10 score and (a) UAFH/LAFH in overall patients, (b) UAFH/LAFH in male patients, (c) AFH in male patients, and (d) AFH/PFH in male patients. Scatter plot is fitted with regression line (red line). The blue bands represent the 95% confidence interval. All data points are shown in the range plotted.

In the study sample overall, K10 score was weakly correlated with UAFH/LAFH (*r* = −0.207, adjusted *P* = 0.048). For male patients, the K10 score was moderately correlated with Bjork's sum (*r* = 0.311, adjusted *P* = 0.012) and AFH (*r* = 0.322, adjusted *P* = 0.012). And K10 score was weakly correlated with FMA (*r* = 0.235), overbite (*r* = −0.238), AFH/PFH (*r* = 0.251), and UAFH/LAFH (*r* = −0.230) at the very edge of significance (adjusted *P* = 0.050). In females, no cephalometric parameters showed correlations with K10 score after adjusting *P* values for multiple testing (Table 4). The correlations between K10 score and AFH, AFH/PFH, and UAFH/LAFH were demonstrated using scatter plots (Figure 5).

The nonadjusted model showed that AFH/PFH was positively related with the K10 score, and UAFH/LAFH was negatively related with the K10 score in multivariate linear regression analysis. After adjustment for age and gender, the association between the K10 score and AFH became significant, and AFH/PFH became insignificant. For each 1 mm increase in AFH, the score of K10 increases 0.147 (*B* = 0.147, 95% CI = 0.012 ~ 0.282). For each 0.1 decrease in UAFH/LAFH, the score of K10 increases 1.4923 (*B* = −14.923, 95% CI = −27.639 ~ −2.206) (Table 5).

## 4. Discussion

In this study, specific craniomaxillofacial features were correlated with psychological distress in adult pretreatment orthodontic patients among male patients. Male patients with a hyperdivergent pattern and open bite were more likely to be under psychological distress. Meanwhile, weak and moderate correlations were observed between K10 score and cephalometric parameters in male and female patients. Therefore, the null hypothesis was rejected.

Previous studies demonstrated that 15% to 37% of orthodontic patients suffer from a certain degree of psychological distress, and around 20% of orthognathic and orthodontic patients show mental instability, introversion, and anxiety [11, 25]. In the current study, we found 25.26% patients had psychological distress, which was consistent with previous studies. However, different psychological and craniomaxillofacial morphological evaluation tools could affect the final results [11]. We chose the K10 scale, which has been widely used to assess psychological distress for patients in dental and other medical fields and has been demonstrated to have good validity and reliability in various subject samples thanks to its ease of use for both respondents and researchers [15, 26–30].

The K10 scale has been extensively used for clinical screening and assessment of efficacy in mental health services. However, several different cut-offs have been validated, depending on the subjects, the measurement method, and the objective of the study [16]. Vasiliadis et al. [31] used the Diagnostic and Statistical Manual of Mental Disorders (DSM) as a criterion measure, and a cutoff of 19 was suggested to balance sensitivity (0.794) and specificity (0.664) for minor psychological distress. Therefore, the threshold of K10 score equal to 20 was considered to be appropriate to distinguish between cases and noncases of psychological distress. Using a threshold of 20 to identify cases of psychological distress, our previous study and other studies has found differences in psychological distress among orthodontic patients, TMD patients, and general population [15, 32, 33]. Since the current study does not determine the most appropriate cut-off value for pretreatment orthodontic patients, the K10 threshold in this study was established based on these cross-sectional studies in the Chinese population.

In this study, about 3% of patients had a K10 score above 30, indicating they were under severe psychological distress [34, 35]. Kessler et al. [36] suggested that about 6% of Americans may suffer from severe mental disorder, and this result was also supported by other studies [37]. The lack of data on orthodontic patients with severe psychological distress so far has limited the clinical management to these patients. The inclusion of the easy-to-use psychological scales in clinical research would be an important step towards addressing this important issue.

The prevalence of psychological distress was not related to gender or age distribution in this study, which was similar to the findings from other studies in Chinese populations [38, 39]. Some studies have suggested that female and young people are more likely to suffer psychological distress [40, 41]. The reason for the varying results might be related to different subjects' characteristics and research backgrounds. Since there are few surveys with large sample sizes on the prevalence of psychological distress among pretreatment orthodontic patients, further research is still needed.

In the measurement of hard tissue structure, differences of AFH and overbite between the psychological distress and no-distress groups among male patients were found. Increased AFH revealed significant increases in the male psychological distress group, suggesting a trend of facial growth in the vertical dimension. Meanwhile, there were higher proportions of hyperdivergent patients in the psychologically distressed group, which also confirmed that they had a larger facial height. Some studies suggested that occlusal factors may be a factor in patients' psychological status and that malocclusion can impair patients' oral health-related quality of life. In this study, we identified reduced overbite and open bite as specific craniomaxillofacial features in psychologically distressed patients. Psychological discomfort and disability are reported much more frequently among patients with severe malocclusion than in normocclusive people [42, 43]. Limited chewing efficiency and unsatisfactory facial appearance might be the causes of psychological distress in patients with malocclusion [43]. At the same time, other studies have revealed that open bite is related to the degree of FMA [44, 45], which is in line with the present study. Therefore, we suggest that patients with psychological distress display a trend in both malocclusion and facial height changes.

Most previous studies evaluated craniomaxillofacial morphology through self-perception and craniofacial photography, which may compromise the accuracy and stability of the assessment. The measurement tools applied in this study, lateral cephalograms, have been recognized as one of the most reliable and repeatable methods to evaluate dental, skeletal, and soft tissue structures in practice [19, 46]. To the best of our knowledge, we evaluated hard tissue and soft tissue structure together for the first time compared with other similar studies, which made the measurement of craniomaxillofacial features more comprehensive and reliable.

Some researchers suggested that disharmonious facial proportions, especially of the lower anterior facial proportion, could lead to an obvious decline in aesthetic self-appraisal [47]. In this study, UAFH/LAFH showed negative correlation with K10 score, indicating a larger lower anterior facial proportion in patients with psychological distress. Therefore, we expect that the larger lower anterior facial proportion is more likely to affect the psychological distress status of orthodontic patients.

Rusanen et al. reported that females with malocclusion and dentofacial deformities show negative psycho-emotion more commonly than males [43]. Another study suggested that females express less tolerance and satisfaction for unattractive dentition than males and hence have a greater desire for orthodontic treatment [48]. However, the current study showed no significant relationship between psychological distress and craniomaxillofacial morphology in females.

One explanation for this negative result may the type of measurements taken. Female self-perceived facial attractiveness has been related to facial width, periorbital region, and nose ridge area shape, as assessed using 3-dimensional facial surface data or frontal-profile craniofacial photography [49, 50]. Cephalograms do not provide the means to evaluate these facial profile characteristics precisely and therefore may explain why such differences between the two groups were not observed. Another explanation may be that females were divided into two groups by other uncontrolled confounding variables such as dental aesthetics, occlusal function, periodontal or mucosal health status, and psychosocial impacts [2, 51], which in distressed female patients have much stronger effects. These factors may weakly related to the measured parameters, so the craniomaxillofacial morphological differences cannot be observed. In future study, the strict control of various confounding factors may improve estimations of the psychological effect of craniomaxillofacial features in female patients. Also, facial morphological differences may not be the main reason for psychological distress in female patients. The effects that other aspects such as oral function, occlusal stability, and smile aesthetics have on psychological distress status might be given more attention by orthodontists [52, 53].

Because the psychological distress may lead to decreased satisfaction with the treatment effects, it is necessary for orthodontists to pay attention to the potential psychological problems of patients in the diagnosis and treatment processes. The present study and other studies demonstrated that psychological distress was commonly detected among pretreatment orthodontic patients. At present, a large number of studies have focused on the psychological status of orthodontic patients from multiple aspects (such as occlusal function and oral-related health), although very few studies have been longitudinal. At the same time, although the influence of facial aesthetics on psychological status has been confirmed by many studies, few studies have explored the relationship between craniomaxillofacial morphology and psychological status in orthodontic patients, especially in pretreatment orthodontic patients.

Cephalometric measurement is a necessary diagnostic process for each patient before treatment. This article explores the relationship between patient profile and psychological state by using measurement analysis tools that are very common in practice. And this will help orthodontists to consider the potential psychological distress of patients with characteristic craniomaxillofacial morphology, so as to avoid possible negative effects on the treatment process due to psychological distress.

Meanwhile, this study suggested that some craniomaxillofacial features might be the more significant factors influencing psychological distress status among orthodontic patients, and sex-specific difference should be considered. This study could provide hints and insights for the rare but important longitudinal studies in this field. In some cases of orthodontic treatment, malocclusion and the proportion of the lower face will change significantly before and after treatment. However, the impact of this change on the patient's psychological state is still unknown. Orthodontists should be aware of the possible influence of changes in craniomaxillofacial morphology on a patient's facial aesthetics and psychological status.

The current study had some limitations. First, patients participated the research voluntarily and the exclusion of patients who refused to cooperate could induce bias, limiting the extrapolation of the conclusions. Second, various confounding factors such as patient economic status, social background, parafunctional habits, dental status, and periodontal status were not controlled in this study. Further research is required to exclude the potential effects of these factors. Third, in this study, no further classification was made for the severity of the patient's psychological distress, and the relationship between craniomaxillofacial morphological features with severity of psychological distress has not been validated. Fourth, the selection of the threshold of K10 could affect the stability of the final results. In future studies, using other standard questionnaires concurrently (such as the 9-item Patient Health Questionnaire (PHQ-9) and the 7-item Generalized Anxiety Disorder (GAD-7)) might allow better determination of the anxiety and depression of the patients. Fifth, the Bland-Altman plots showed that there might be random errors for the digital tracing. Although the examiner tried to minimize the potential measurement errors in formal measurements, random errors in the process of cephalometric analysis still cannot be completely avoided, which might affect the reliability of the results in this study. Last, our findings may only be relevant to Chinese populations because of ethnic differences.

## 5. Conclusion

In the studied sample, one-quarter of adult pretreatment orthodontic patients were experiencing psychological distress. Psychological distress was mainly correlated with hyperdivergent pattern, open bite, and larger lower anterior facial height proportion in pretreatment orthodontic patients. Orthodontists should be aware of the possible underlying psychological distress in patients with specific craniomaxillofacial features. Clinical assessment of psychological distress may need to take into account gender differences in patients.

## Figures and Tables

**Table 1:** Gender, age, and K10 score characteristics of patients.

		No psychological distress	Psychological distress	*P*
Gender	Male, *n* (%)	73 (38.42)	22 (11.58)	0.617
	Female, *n* (%)	69 (36.32)	26 (13.68)	
Age	Mean ± SD	25.75 ± 6.82	24.24 ± 5.36	0.302
	Median (IQR)	24.29 (19.92, 29.62)	22.88 (19.39, 27.30)	
K10 score	Mean ± SD	12.84 ± 3.00	25.54 ± 4.51	0.000^∗^
	Median (IQR)	12.00 (10.00, 15.00)	25.00 (21.25, 29.00)	

Chi-square test and Mann-Whitney *U* test are used. ^∗^*P* < 0.05. SD: standard deviation; IQR: interquartile range.

**Table 2:** Differences of cephalometric parameters in male patients with and without psychological distress (*n* = 95).

Cephalometric parameters	No psychological distress	Psychological distress	*t*	*P* value	Adjusted *P* value
*Hard tissue*					
ANB (°)	2.79 ± 3.76	3.13 ± 4.26	-0.356	0.723	0.771
FMA (°)	21.57 ± 6.15	25.03 ± 5.08	-2.404	0.018	0.112
Saddle angle (°)	123.44 ± 4.70	120.60 ± 5.80	2.351	0.021	0.112
Articular angle (°)	149.66 ± 6.50	152.35 ± 5.91	-1.736	0.086	0.172
Gonial angle (°)	115.96 ± 7.65	119.32 ± 6.12	-1.882	0.063	0.144
Bjork's sum (°)	389.06 ± 6.50	392.26 ± 5.84	-2.070	0.041	0.131
Ramus height (mm)	51.91 ± 5.02	51.66 ± 4.55	0.207	0.837	0.864
Mandibular body length (mm)	73.88 ± 4.62	72.58 ± 4.92	1.135	0.259	0.440
Anterior cranial base length (mm)	66.32 ± 3.05	65.00 ± 2.88	1.804	0.075	0.161
Posterior cranial base length (mm)	37.13 ± 3.02	37.74 ± 2.75	-0.846	0.399	0.581
AFH (mm)	120.97 ± 6.87	126.62 ± 4.32	-4.621	0.000	0.001^∗^
PFH (mm)	85.88 ± 6.26	86.62 ± 5.41	-0.502	0.617	0.681
LAFH (mm)	66.72 ± 6.17	69.89 ± 5.24	-2.181	0.032	0.131
UAFH (mm)	54.09 ± 2.97	54.83 ± 2.75	-1.038	0.302	0.478
Wits appraisal (mm)	−0.02 ± 5.22	−1.63 ± 6.65	1.125	0.261	0.440
Overjet (mm)	3.14 ± 3.53	2.1 ± 3.89	1.884	0.060	0.144
Overbite (mm)	2.75 ± 1.92	1.21 ± 1.84	3.332	0.001	0.016^∗^
AFH/PFH	1.41 ± 0.11	1.47 ± 0.10	-2.054	0.043	0.131
UAFH/LAFH	0.82 ± 0.07	0.79 ± 0.08	1.506	0.135	0.254
*Soft tissue*					
Facial convexity (°)	168.15 ± 7.07	167.17 ± 7.49	0.561	0.576	0.681
Nasolabial angle (°)	83.20 ± 12.35	84.78 ± 13.24	-0.518	0.606	0.681
Nasal prominence (°)	16.57 ± 2.18	16.23 ± 2.05	0.646	0.520	0.666
Upper lip length (mm)	23.26 ± 2.52	25.11 ± 2.42	-3.052	0.003	0.032^∗^
Lower lip length (mm)	17.18 ± 3.04	18.70 ± 2.55	-2.124	0.036	0.131
Lower lip to E plane (mm)	1.76 ± 3.52	2.20 ± 3.50	-0.518	0.605	0.681
Upper lip to E plane (mm)	−0.36 ± 3.39	0.47 ± 3.38	-1.011	0.314	0.478
Lower lip thickness (mm)	14.74 ± 1.78	14.33 ± 2.81	0.651	0.520	0.666
Upper lip thickness (mm)	13.18 ± 1.86	13.67 ± 3.02	-0.723	0.476	0.662
Soft tissue chin thickness (mm)	11.71 ± 2.21	10.51 ± 1.69	2.358	0.020	0.112
Sn-Me' (mm)	74.93 ± 6.71	78.06 ± 5.39	-1.999	0.049	0.131
N'-Sn (mm)	59.08 ± 3.98	59.17 ± 3.10	-0.095	0.925	0.925
N'-Sn/Sn-Me'	0.79 ± 0.07	0.76 ± 0.06	2.009	0.047	0.131

Cephalometric parameter measurements are expressed as mean ± standard deviation. Mann-Whitney *U* test and independent sample *t*-test are used. ^∗^*P* < 0.05. FMA: Frankfort-mandibular plane angle; AFH: anterior facial height; PFH: posterior facial height; UAFH: upper anterior facial height; LAFH: lower anterior facial height.

**Table 3:** Cephalometric parameter stratification analysis of patients with and without psychological distress.

Cephalometric parameter stratification	Male (*n* = 95)				Female (*n* = 95)			
	No psychological distress	Psychological distress	*χ* ^2^	*P*	No psychological distress	Psychological distress	*χ* ^2^	*P*
*ANB*								
Skeletal class I	32	9	0.907	0.625	26	10	0.778	0.750
Skeletal class II	26	10			30	13		
Skeletal class III	15	3			13	3		
*FMA*								
Hypodivergent	40^a^	5^a^	7.622	0.029^∗^	19	11	4.351	0.124
Normodivergent	22^ab^	10^ab^			32	6		
Hyperdivergent	11^b^	7^b^			18	9		
*Overjet*								
Crossbite	14	5	5.520	0.056	14	2	2.675	0.296
Normal	11	8			11	6		
Deep overjet	48	9			44	18		
*Overbite*								
Open bite	15^a^	9^a^	9.374	0.010^∗^	20	6	3.477	0.188
Normal	26^a^	11^a^			26	6		
Deep overbite	32^b^	2^b^			23	14		

Chi-square test and Fisher test are used. Different letters indicate statistically significant differences between the groups after pairwise comparison. ^∗^*P* < 0.05. FMA: Frankfort-mandibular plane angle.

**Table 4:** Correlation between K10 score and cephalometric parameters.

Cephalometric parameters	Overall (*n* = 190)			Male (*n* = 95)			Female (*n* = 95)		
	*P* value	Adjusted *P* value	*r*	*P* value	Adjusted *P* value	*r*	*P* value	Adjusted *P* value	*r*
FMA (°)	0.095	0.228	0.122	0.022	0.050	0.235	0.895	0.895	-0.014
Bjork's sum (°)	0.022	0.088	0.166	0.002	0.012	0.311^∗^	0.560	0.747	0.061
AFH (mm)	0.165	0.291	0.101	0.001	0.012	0.322^∗^	0.883	0.895	0.015
Overbite (mm)	0.530	0.585	-0.046	0.020	0.050	-0.238	0.203	0.406	0.132
AFH/PFH	0.017	0.088	0.173	0.014	0.050	0.251	0.419	0.718	0.084
UAFH/LAFH	0.004	0.048	-0.207^∗^	0.025	0.050	-0.230	0.154	0.370	-0.147

Spearman correlation analysis is used. Only statistically significant results are presented in this table. ^∗^*P* < 0.05. FMA: Frankfort-mandibular plane angle; AFH: anterior facial height; PFH: posterior facial height; UAFH: upper anterior facial height; LAFH: lower anterior facial height.

**Table 5:** Multivariate linear regression analysis between the K10 score and the cephalometric parameters, adjusted for gender and age.

Cephalometric parameters	Nonadjusted		Adjusted model	
	*B* (95% CI)	Adjusted *P* value	*B* (95% CI)	Adjusted *P* value
AFH	0.088	0.146	0.147	0.033^∗^
	(-0.031, 0.208)		(0.012, 0.282)	
AFH/PFH	7.970	0.038^∗^	6.709	0.099
	(0.457, 15.483)		(-1.266, 14.685)	
UAFH/LAFH	-14.955	0.038^∗^	-14.923	0.033^∗^
	(-27.678, -2.231)		(-27.639, -2.206)	

Adjusted model adjusts for age and gender. ^∗^*P* < 0.05. CI: confidence interval; AFH: anterior facial height; PFH: posterior facial height; UAFH: upper anterior facial height; LAFH: lower anterior facial height.

## Data Availability

The data used to support the findings of this study are available from the corresponding author upon request.

## Supplementary Materials

Supplementary Materials Supplement Table 1: differences of cephalometric parameters in patients with and without psychological distress (*n* = 190). Supplement Table 2: differences of cephalometric parameters in female patients with and without psychological distress (*n* = 95).
