# Supplementary material for: Do Specific Craniomaxillofacial Features Correlate with Psychological Distress in Adult Pretreatment Orthodontic Patients? A Cephalometric Study
Source: Dis Markers. 2022 May 5;2022:9694413. doi: 10.1155/2022/9694413 (PMC9098301; doi:10.1155/2022/9694413)
Supplement: Supplementary Materials — Supplement Table 1: differences of cephalometric parameters in patients with and without psychological distress (n = 190). Supplement Table 2: differences of cephalometric parameters in female patients with and without psychological distress (n = 95). [file 9694413.f1.docx]

## Supplemental material

**Supplemental table 1** Differences of cephalometric parameters in patients with and without psychological distress (n=190)

| Cephalometric parameters | No psychological distress | Psychological distress | t | *P* value | Adjusted *P* value |
| --- | --- | --- | --- | --- | --- |
| **Hard tissue** |  |  |  |  |  |
| ANB /(°) | 2.86±3.67 | 3.48±3.73 | -0.996 | 0.321 | 0.790 |
| FMA /(°) | 23.09±6.32 | 24.79±6.40 | -1.611 | 0.109 | 0.409 |
| Saddle angle /(°) | 123.05±5.04 | 122.70±5.36 | 0.413 | 0.680 | 0.912 |
| Articular angle /(°) | 151.06±6.58 | 151.65±6.15 | -0.541 | 0.589 | 0.898 |
| Gonial angle /(°) | 117.01±7.06 | 118.80±7.33 | -1.504 | 0.134 | 0.429 |
| Bjork's sum /(°) | 391.13±6.93 | 393.15±7.11 | -1.735 | 0.084 | 0.409 |
| Ramus height /mm | 48.79±5.79 | 49.04±5.49 | -0.263 | 0.793 | 0.912 |
| Mandibular Body length /mm | 71.84±4.82 | 71.31±4.95 | 0.658 | 0.511 | 0.860 |
| Anterior cranial base length /mm | 65.03±3.23 | 64.01±3.31 | 1.891 | 0.060 | 0.409 |
| Posterior cranial base length /mm | 35.42±3.66 | 35.25±3.56 | 0.285 | 0.776 | 0.912 |
| AFH /mm | 118.04±7.71 | 120.99±7.67 | -2.301 | 0.022 | 0.409 |
| PFH /mm | 81.47±7.83 | 81.63±7.21 | -0.131 | 0.896 | 0.925 |
| LAFH /mm | 66.04±6.03 | 67.74±6.51 | -1.659 | 0.099 | 0.409 |
| UAFH /mm | 53.65±2.95 | 53.65±3.34 | 0.017 | 0.987 | 0.987 |
| Wits appraisal /mm | -0.09±4.84 | -0.31±5.84 | 0.256 | 0.798 | 0.912 |
| Overjet /mm | 3.12±3.33 | 3.28±3.32 | -0.284 | 0.776 | 0.912 |
| Overbite /mm | 2.48±2.08 | 2.24±2.26 | 0.681 | 0.497 | 0.861 |
| AFH / PFH | 1.46±0.12 | 1.49±0.12 | -1.583 | 0.115 | 0.409 |
| UAFH / LAFH | 0.82±0.07 | 0.80±0.07 | 1.707 | 0.089 | 0.409 |
| **Soft tissue** |  |  |  |  |  |
| Facial convexity /(°) | 168.22±6.78 | 167.32±7.26 | 0.780 | 0.436 | 0.861 |
| Nasolabial angle /(°) | 83.73±12.81 | 85.00±12.52 | -0.598 | 0.551 | 0.882 |
| Nasal prominence /(°) | 16.52±2.02 | 16.24±1.69 | 0.843 | 0.400 | 0.861 |
| Upper lip length /mm | 22.72±2.68 | 23.62±2.69 | -2.016 | 0.045 | 0.409 |
| Lower lip length /mm | 16.72±2.88 | 16.80±2.85 | -0.170 | 0.866 | 0.924 |
| Lower lip to E plane /mm | 1.57±3.24 | 1.40±3.16 | 0.318 | 0.751 | 0.912 |
| Upper lip to E plane /mm | -0.35±3.17 | 0.06±2.90 | -0.796 | 0.427 | 0.861 |
| Lower lip thickness /mm | 13.89±2.01 | 13.95±2.34 | -0.162 | 0.871 | 0.925 |
| Upper lip thickness /mm | 12.16±2.26 | 12.43±2.56 | -0.678 | 0.499 | 0.861 |
| Soft tissue chin thickness /mm | 11.84±2.20 | 11.24±2.09 | 1.662 | 0.098 | 0.409 |
| Sn-Me’ /mm | 73.05±6.53 | 74.36±6.21 | -1.223 | 0.223 | 0.649 |
| N’-Sn /mm | 57.89±3.83 | 58.12±3.33 | -0.375 | 0.708 | 0.912 |
| N’-Sn / Sn-Me’ | 0.80±0.07 | 0.79±0.06 | 1.079 | 0.282 | 0.752 |

Cephalometric parameter measurements are expressed as mean ± standard deviation. Mann Whitney U test and independent samples t-test are used.

FMA, Frankfort-mandibular plane angle; AFH, anterior facial height; PFH, posterior facial height; UAFH, upper anterior facial height; LAFH, lower anterior facial height.

**Supplemental table 2** Differences of cephalometric parameters in female patients with and without psychological distress (n=95)

| Cephalometric parameters | No psychological distress | Psychological distress | t | *P* | Adjusted *P* value |
| --- | --- | --- | --- | --- | --- |
| **Hard tissue** |  |  |  |  |  |
| ANB /(°) | 3.02±3.61 | 3.77±3.26 | -0.928 | 0.356 | 0.929 |
| FMA /(°) | 24.91±6.38 | 24.59±7.44 | 0.208 | 0.836 | 0.932 |
| Saddle angle /(°) | 122.63±5.40 | 124.48±4.32 | -1.567 | 0.121 | 0.896 |
| Articular angle /(°) | 152.61±6.39 | 151.05±6.41 | 1.056 | 0.294 | 0.929 |
| Gonial angle /(°) | 118.27±6.39 | 118.37±8.32 | -0.056 | 0.956 | 0.956 |
| Bjork's sum /(°) | 393.32±6.72 | 393.90±8.07 | -0.356 | 0.723 | 0.929 |
| Ramus height /mm | 45.44±4.71 | 46.83±5.31 | -1.242 | 0.217 | 0.929 |
| Mandibular Body length /mm | 69.65±4.06 | 70.23±4.81 | -0.594 | 0.554 | 0.929 |
| Anterior cranial base length /mm | 63.63±2.89 | 63.17±3.46 | 0.659 | 0.512 | 0.929 |
| Posterior cranial base length /mm | 33.68±3.41 | 33.15±2.73 | 0.707 | 0.482 | 0.929 |
| AFH /mm | 115.10±7.36 | 116.24±6.61 | -0.690 | 0.492 | 0.929 |
| PFH /mm | 76.79±6.54 | 77.42±5.73 | -0.427 | 0.671 | 0.929 |
| LAFH /mm | 65.31±5.82 | 65.92±7.01 | -0.429 | 0.669 | 0.929 |
| UAFH /mm | 53.19±2.86 | 52.65±3.52 | 0.780 | 0.438 | 0.929 |
| Wits appraisal /mm | -0.13±4.44 | 0.80±4.92 | -0.883 | 0.379 | 0.929 |
| Overjet /mm | 3.1±3.14 | 4.28±2.41 | -1.733 | 0.086 | 0.896 |
| Overbite /mm | 2.11±2.31 | 3.11±2.24 | -1.901 | 0.060 | 0.896 |
| AFH / PFH | 1.50±0.12 | 1.51±0.14 | -0.197 | 0.845 | 0.932 |
| UAFH / LAFH | 0.82±0.07 | 0.80±0.07 | 0.945 | 0.347 | 0.929 |
| **Soft tissue** |  |  |  |  |  |
| Facial convexity /(°) | 168.3±6.50 | 167.45±7.20 | 0.550 | 0.584 | 0.929 |
| Nasolabial angle /(°) | 84.29±13.34 | 85.19±12.13 | -0.299 | 0.766 | 0.932 |
| Nasal prominence /(°) | 16.46±1.84 | 16.25±1.37 | 0.521 | 0.603 | 0.929 |
| Upper lip length /mm | 22.15±2.74 | 22.36±2.26 | -0.351 | 0.726 | 0.929 |
| Lower lip length /mm | 16.22±2.63 | 15.19±1.98 | 1.814 | 0.073 | 0.896 |
| Lower lip to E plane /mm | 1.38±2.94 | 0.73±2.73 | 0.983 | 0.328 | 0.929 |
| Upper lip to E plane /mm | -0.33±2.95 | -0.28±2.44 | -0.083 | 0.934 | 0.956 |
| Lower lip thickness /mm | 12.99±1.85 | 13.63±1.86 | -1.488 | 0.140 | 0.896 |
| Upper lip thickness /mm | 11.09±2.15 | 11.38±1.48 | -0.626 | 0.533 | 0.929 |
| Soft tissue chin thickness /mm | 11.98±2.21 | 11.86±2.23 | 0.242 | 0.810 | 0.932 |
| Sn-Me’ /mm | 71.06±5.73 | 71.24±5.10 | -0.141 | 0.888 | 0.947 |
| N’-Sn /mm | 56.63±3.24 | 57.24±3.31 | -0.808 | 0.421 | 0.929 |
| N’-Sn / Sn-Me’ | 0.80±0.06 | 0.81±0.05 | -0.373 | 0.710 | 0.929 |

Cephalometric parameter measurements are expressed as mean ± standard deviation. Mann Whitney U test and independent samples t-test are used.

FMA, Frankfort-mandibular plane angle; AFH, anterior facial height; PFH, posterior facial height; UAFH, upper anterior facial height; LAFH, lower anterior facial height.
